# Effects of Carbon Doping on the Structural, Energetic, and Electronic Properties of Intrinsic Defects in α‑Al_2_O_3_

**DOI:** 10.1021/acsomega.5c05808

**Published:** 2025-12-05

**Authors:** Fengai Zhao, Hongyan Wang, Fang Wang, Ye Tian

**Affiliations:** † 56711 Southwest Jiaotong University, School of Physical Science and Technology Chengdu, Sichuan 610031, China; ‡ China Academy of Engineering Physics Research Center of Laser Fusion Mianyang, Sichuan 621900, China

## Abstract

The atomic-scale mechanisms governing the carbon doping effects in α-Al_2_O_3_ remain incompletely understood. Using density functional theory (DFT), we systematically investigate how carbon substitution modulates the structural, energetic, and electronic properties of α-Al_2_O_3_ with intrinsic defects. The findings reveal that structural distortion is strongly site-dependent. C substitution at oxygen sites (C_O_) induces significantly greater lattice deformation than aluminum-site substitution (C_Al_). Average distances from defect positions to neighboring atom analyses show that C doping preferentially perturbs the O atom distances in V_Al_/Al_i_ systems, while dominantly affecting Al-atom distances in V_O_/O_i_ systems. Furthermore, formation energies of intrinsic defects decrease by up to 35% with C doping under both O-rich and O-poor conditions. C_O_ doping most effectively stabilizes vacancies/interstitials, while C_Al_ preferentially lowers Frenkel pair energies. Aluminum vacancies (V_Al_) exhibit the lowest formation energy. Additionally, C doping introduces midgap states through C 2p and O 2p hybridization, narrowing band gaps from 6.16 eV (pristine) to 1.60–4.45 eV. This work provides crucial insights into how carbon doping modulates defect formation and local structure in α-Al_2_O_3_, offering valuable guidance for the strategic design of alumina-based materials with tailored properties through fine-tuning defect engineering.

## Graphical abstract

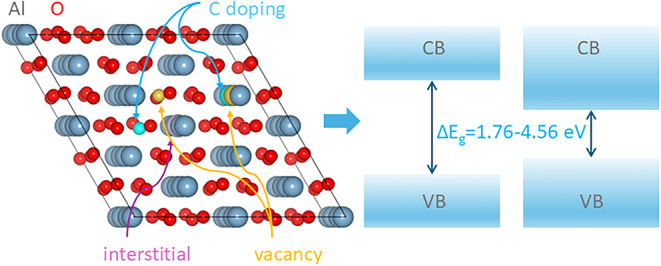

## 1 Introduction

Aluminum oxide has long been recognized as a thermoluminescent (TL) material, but its use in dosimetric applications has historically been limited by low sensitivity.^1–3^ A highly sensitive TL dosimeter, the α-Al_2_O_3_:C crystal, was discovered in 1990. This material exhibits excellent TL properties, including: (i) high sensitivity—approximately 40–60 times greater than that of LiF: Mg, Ti (at a heating rate of 4 K/s); (ii) a glow curve featuring a prominent peak at 463 K; (iii) low background signal and dose threshold (detectable down to 0.1 μGy under nitrogen flow); (iv) minimal fading during dark storage (<5% per year); (v) good reproducibility (<2%) and reusability without annealing; and (vi) a wide linear dose range from 10^–7^ to 10 Gy.^4–6^ However, despite three decades of research since its discovery, two key challenges persist: (i) the precise mechanism by which carbon doping enhances TL efficiency remains debated and (ii) the interplay between carbon dopants and intrinsic defects (*e*.*g*., vacancies, interstitials) in α-Al_2_O_3_—an essential factor governing its luminescent properties—is poorly understood.

Current understanding of α-Al_2_O_3_:C relies on two competing hypotheses: Akselrod et al. proposed that carbon substitutes for Al^3+^ (C^2+^→Al^3+^), creating hole-trapping F^+^ centers to compensate charge.^4^ The heterovalent substitution of C^2+^ for Al^3+^ creates a net negative charge imbalance. To maintain charge neutrality, positively charged defects must form during crystal growth. F^+^ centers (oxygen vacancies with one trapped electron, denoted as V_O_^•^) act as intrinsic charge compensators.^4,7^ While F^+^ centers are metastable in pure α-Al_2_O_3_, carbon doping stabilizes them by localizing electrons near C_Al_ sites, facilitating hole trapping essential for TL. However, Yang et al. recently suggested C occupies O sites, directly modulating carrier concentrations.^5^ Experimental studies report conflicting roles for carbon: some attribute TL enhancement to C-induced defect stabilization,^5^ while others argue C primarily aids crystal growth rather than TL mechanisms.^8^ This ambiguity stems from limited atomic-scale insights into how carbon doping interacts with intrinsic defects (*e*.*g*., vacancies, Frenkel pairs) to alter structural, energetic, and electronic properties—critical for optimizing TL performance.

Recent theoretical advances, such as DFT studies by Zhu et al.^9^ and Ao et al.,^10^ have explored carbon substitution preferences (Al vs O sites) under oxygen-rich/aluminum-rich conditions but have not systematically investigated the combined effects of carbon doping and multiple intrinsic defects. For instance, how C doping at Al vs O sites affects the formation energy of aluminum interstitials or oxygen vacancies. Whether defect-dopant interactions stabilize or destabilize TL-active centers. These questions remain unanswered, hindering targeted design of next-generation α-Al_2_O_3_:C dosimeters.

In this work, we address these gaps by performing a comprehensive DFT study of carbon-doped α-Al_2_O_3_ containing six intrinsic defects (aluminum interstitial (Al_i_), oxygen interstitial (O_i_), aluminum vacancy (V_Al_), oxygen vacancy (V_O_), aluminum Frenkel pair (FP_Al_), and oxygen Frenkel pair (FP_O_)). We systematically analyze: (1) how C substitution at Al vs O sites impacts defect formation energies and structural distortions and (2) electronic property changes (band gaps, defect states) that govern TL efficiency. By bridging atomic-scale simulations with experimental observables, we aim to provide a mechanistic framework for optimizing α-Al_2_O_3_:C’s performance, thereby advancing its application in high-sensitivity radiation detection.

## 2 Computational Details

All calculations were performed by using the Vienna *Ab Initio* Simulation Package (VASP). The exchange-correlation function was treated within the local-density approximation (LDA). The projector augmented wave (PAW) method was used to describe the electron–ion interactions.^11,12^ The valence electron configurations for the PAW potentials were Al: 3s^2^3p^1^, O: 2s^2^2p^4^, and C: 2s^2^2p^2^. The α-Al_2_O_3_ crystal (hexagonal structure, *R*3̅*c* space group) was modeled using a 120-atom 2 × 2 × 1 supercell. After convergence testing, a plane-wave kinetic energy cutoff of 500 eV and a 2 × 2 × 2 Monkhorst–Pack *k*-point mesh were employed for Brillouin zone sampling.

To investigate carbon doping effects on structural and energetic properties of defective α-Al_2_O_3_, we introduced six intrinsic defects (Al_i_, O_i_, V_Al_, V_O_, FP_Al_, and FP_O_) into the pristine lattice. Additionally, a C substituting for an Al atom in α-Al_2_O_3_ (α-Al_2_O_3_+C_Al_) and α-Al_2_O_3_+C_Al_ with each intrinsic defect, *i*.*e*., α-Al_2_O_3_+C_Al_:Al_i_, α-Al_2_O_3_+C_Al_:O_i_, α-Al_2_O_3_+C_Al_:V_Al_, α-Al_2_O_3_+C_Al_:V_O_, α-Al_2_O_3_+C_Al_:FP_Al_, and α-Al_2_O_3_+C_Al_:FP_O_, were also calculated. Similarly, a C replacing an O atom (α-Al_2_O_3_+C_O_) and α-Al_2_O_3_+C_O_ with each intrinsic defect, *i*.*e*., α-Al_2_O_3_+C_O_:Al_i_, α-Al_2_O_3_+C_O_:O_i_, α-Al_2_O_3_+C_O_:V_Al_, α-Al_2_O_3_+C_O_:V_O_, α-Al_2_O_3_+C_O_:FP_Al_, and α-Al_2_O_3_+C_O_:FP_O_, were also considered. Corresponding geometric configurations are listed in Figure 1. It is noted that our DFT calculations use a neutral defect model, where we do not explicitly include charged defects (*e*.*g*., F^+^) or adjust chemical potentials for ionized states. Instead, we implicitly account for charge balance by considering intrinsic defects (*e*.*g*., V_Al_, and O_i_) that naturally compensate for C doping. While explicit consideration of charged defects (*e*.*g*., F^+^) would refine the model, our neutral approximation is valid for studying relative defect stabilities and trends, as the primary effect of charge compensation is captured by the inclusion of intrinsic defects in the calculation. Neglecting explicit charge states may slightly affect absolute formation energy values but does not alter the qualitative conclusions.

**Figure 1:**
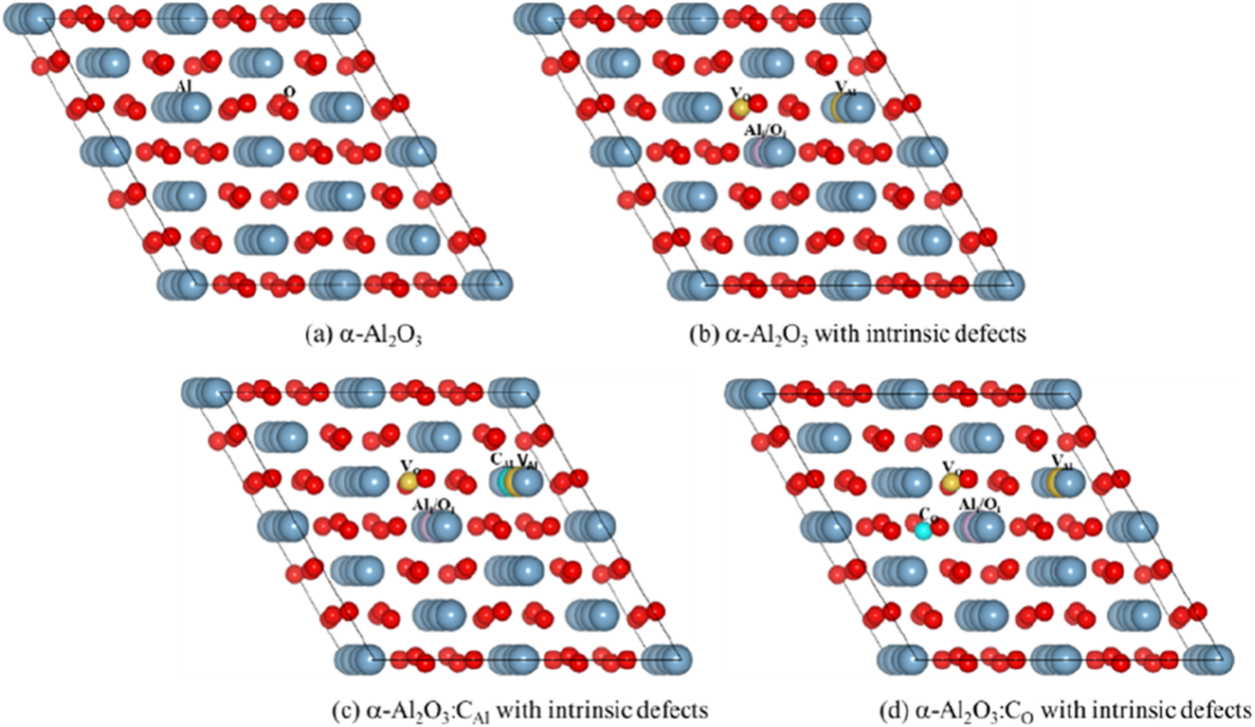
Schematic views of (a) α-Al_2_O_3_, (b) α-Al_2_O_3_ with intrinsic defects including aluminum interstitial (Al_i_), oxygen interstitial (O_i_), aluminum vacancy (V_Al_), oxygen vacancy (V_O_), aluminum Frenkel pair (FP_Al_), and oxygen Frenkel pair (FP_O_), (c) a C substituting for an Al atom in α-Al_2_O_3_ with each intrinsic defect, and (d) a C substituting for an O atom in α-Al_2_O_3_ with each intrinsic defect. The gray and red spheres represent Al and O atoms, respectively. Yellow spheres of different sizes denote V_Al_ and V_O_ defects, while blue spheres of different sizes represent C_Al_ and C_O_ defects. The purple spheres correspond to the Al_i_ and O_i_ defects.

## 3 Results and Discussion

### 3.1 Structural Properties of Intrinsic Defects in Undoped and C-Doped α-Al_2_O_3_

The optimized lattice constants for α-Al_2_O_3_ are presented in Table 1 alongside the available experimental and theoretical data. Table 1 also summarizes optimized lattice parameters and fractional volume changes (Δ*V*/*V*_0_) relative to those of pristine α-Al_2_O_3_ for both defect-free and defective systems with/without carbon doping. For comparison, the lattice parameters and ΔV/V_0_ for α-Al_2_O_3_ with intrinsic defects without C doping are first illustrated. For the ideal crystal, our LDA-calculated *a*-axis (4.73 Å) is slightly smaller than experimental measurements of 4.77 Å^13^ and 4.76 Å^14^ due to LDA’s well-known underestimation of bond lengths. However, the trend matches the experimental data: C–Al substitution (α-Al_2_O_3_:C_Al_) increases *c*-axis length (12.95 Å vs 12.90 Å defect-free), while C–O substitution (α-Al_2_O_3_:C_O_) slightly increases *a*-axis (4.74 Å). These distortions align with experimental observations of the lattice strain in doped α-Al_2_O_3_. For example, Yang et al. reported increased lattice parameters with carbon doping.^5^

**Table 1:** Optimized Lattice Parameters and Volume Changes (Δ*V*/*V*_0_) for Defect-Free and Defective α-Al_2_O_3_ ^a^

	phase	(*a*, *b*, *c*)	(α, β, γ)	Δ*V*/*V*_0_ (%)
α-Al_2_O_3_	defect-free	(4.73,4.73,12.90)	(90°, 90°, 120°)	
	Exp.^13^	(4.77,4.77,13.01)	(90°, 90°, 120°)	
	Cal.^15^	(4.80,4.80,13.10)	(90°, 90°, 120°)	
	Cal.^9^	(4.70,4.70,12.84)		
	Al_i_	(4.78,4.78, 12.99)	(90°, 90°, 120°)	2.5
	O_i_	(4.75, 4.75, 12.90)	(90°, 90°,120°)	0.7
	V_Al_	(4.74,4.74,12.93)	(90°, 90°, 120°)	0.3
	V_O_	(4.73,4.73,12.90)	(90.04°, 89.96°, 119.94°)	–0.1
	FP_Al_	(4.74,4.74, 13.05)	(90°, 90°, 120°)	1.1
	FP_O_	(4.76, 4.75,12.93)	(90.47°, 89.70°, 120.02°)	1.2
α-Al_2_O_3_:C_Al_	defect-free	(4.73,4.73,12.95)	(90°, 90°, 120°)	–0.1
	Al_i_	(4.76,4.76,13.09)	(90°, 90°, 120°)	2.4
	O_i_	(4.76,4.76,12.88)	(90°, 90°, 120°)	0.6
	V_Al_	(4.73,4.73,12.97)	(90°, 90°, 120°)	0.3
	V_O_	(4.72,4.72,12.95)	(90.01°, 90.04°, 119.90°)	–0.1
	FP_Al_	(4.73,4.73,13.10)	(90°, 90°, 120°)	1.5
	FP_O_	(4.76,4.76,12.97)	(89.86°, 90.07°, 120.09°)	1.5
α-Al_2_O_3_:C_O_	defect-free	(4.74,4.74,12.95)	(89.71°, 90.29°, 120.11°)	0.5
	Al_i_	(4.76,4.76,13.06)	(90.13°, 89.98°, 120.07°)	2.2
	O_i_	(4.76,4.78,12.93)	(89.53°, 90.30°, 120.07°)	1.2
	V_Al_	(4.76,4.72,12.89)	(89.96°, 90.10°, 119.93°)	0.03
	V_O_	(4.76,4.73,12.96)	(89.87°, 90.03°, 120.07°)	0.8
	FP_Al_	(4.75,4.75,12.94)	(90.18°, 90.04°, 119.89°)	0.5
	FP_O_	(4.76,4.76,12.98)	(89.55°, 90.13°, 120.09°)	1.5

a All lengths are given in Å. Δ*V*/*V*_0_: volume changes relative to the defect-free α-Al_2_O_3_.

Among intrinsic defects in pure α-Al_2_O_3_, aluminum interstitials (Al_i_) induce the most significant structural deformation (lattice parameter variation and Δ*V*/*V*_0_), while oxygen vacancies (V_O_) cause the least. Other defects exhibit intermediate deformation magnitudes. Carbon substitution at aluminum sites (C_Al_) in defective systems induces structural deformations similar to those in undoped defective α-Al_2_O_3_. For carbon substitution for oxygen sites (C_O_) in α-Al_2_O_3_ with intrinsic defects, the aluminum interstitial (Al_i_) still produces the greatest deformation, whereas aluminum vacancies (V_Al_) yield the smallest deformation. Compared with C doping at the Al site, the substitution of C for O introduces greater structural distortion in α-Al_2_O_3_ containing intrinsic defects. This difference arises from the larger ionic radius mismatch between C^4+^ and O^2–^ compared to C^4+^ and Al^3+^.

To further explore the effects of C doping on intrinsic defects in α-Al_2_O_3_, average distances between intrinsic defects and neighboring atoms (Al, O) after structural relaxation were examined for both undoped and C-doped systems (Table 2). For α-Al_2_O_3_:V_Al_, the Al vacancy is coordinated by six O atoms at 2.10 Å and four Al atoms at 2.65 Å. Carbon doping reduces the average V_Al_-O distance (d ⟨V_Al_-O⟩) while increasing the V_Al_-Al distance (d ⟨V_Al_-Al⟩). Specifically, the substitution of C for Al sites reduces d ⟨V_Al_-O⟩ by 9.0% and increases d ⟨V_Al_-Al⟩ by 3.8%. These changes are smaller for d ⟨V_Al_-O⟩ and larger for d ⟨V_Al_-Al⟩ compared to C doping at an O site. Akselrod et al. reported that α-Al_2_O_3_:C has a prominent TL peak at 463 K, attributed to the recombination of electrons trapped at V_Al_ and holes at F^+^ centers.^4^ Our structural analysis shows C doping alters V_Al_-O bond lengths (reducing d ⟨V_Al_-O⟩ by 9.0% in C–Al substitution), which could stabilize V_Al_ and enhance electron trapping, explaining the enhanced 463 K peak intensity. In the case of α-Al_2_O_3_:V_O_, the O vacancy is surrounded by four Al atoms at 1.86 Å and 12 O atoms at 2.70 Å. Carbon doping induces the outward relaxation of neighboring Al atoms. When C occupies an Al site, adjacent O atoms relax inward; when C substitutes for an O site, O atoms exhibit slight outward relaxation.

**Table 2:** Average Distance from the Defect Positions to Neighboring Al and O Atoms for α-Al_2_O_3_ with and without C Doping^a^

	d ⟨D_def_-O⟩		d ⟨D_def_-Al⟩
		D_def_ = V_Al_	
α-Al_2_O_3_	2.10		2.65
α-Al_2_O_3_+C_Al_	1.93		2.75
α-Al_2_O_3_+C_O_	1.91		2.70
		D_def_ = V_O_	
α-Al_2_O_3_	2.70		1.86
α-Al_2_O_3_+C_Al_	2.67		2.01
α-Al_2_O_3_+C_O_	2.71		2.06
		D_def_ = Al_i_	
α-Al_2_O_3_	2.19		2.25
α-Al_2_O_3_+C_Al_	1.85		2.31
α-Al_2_O_3_+C_O_	1.93		2.32
		D_def_ = O_i_	
α-Al_2_O_3_	2.14		1.87
α-Al_2_O_3_+C_Al_	2.16		2.07
α-Al_2_O_3_+C_O_	2.48		2.15

a All lengths are in Å.

In α-Al_2_O_3_:Al_i_, the Al interstitial molecule is coordinated by two Al atoms at 2.25 Å and six O atoms at 2.19 Å. C doping (at Al or O sites) decreases the distance between Al_i_ and neighboring O atoms but increases the distance to the surrounding Al atoms. For α-Al_2_O_3_:O_i_, the interstitial O atom exhibits average distances d ⟨O_i_-O⟩ = 2.14 Å and d ⟨O_i_-Al⟩ = 1.87 Å. Carbon substitution for an O site induces greater outward relaxation of neighboring Al and O atoms than substitution for an Al site. Notably, C doping more significantly affects distances to the O neighbors than to the Al neighbors in V_Al_ and Al_i_ systems. Conversely, for V_O_ and O_i_ systems, doping has a larger effect on the distances to Al neighbors than to O neighbors.

### 3.2 Effects of C Doping on the Stability of α-Al_2_O_3_ with Intrinsic Defects

To investigate the effects of C doping on the stability of intrinsic defects in α-Al_2_O_3_, we calculated the formation energies of these defects in both pristine and C-doped α-Al_2_O_3_. The calculations were based on the formalism of Zhang and Northrup,^16^ utilizing the total energies obtained from defective supercells. The formation energy (*E*_form_) for a defect is given by $E_{form}=E_{tot}\left(defect\right)-E_{tot}\left(p\mathrm{erf}ect\right)+\sum_{i}n_{i}\mu_{i}$ , where *E*_tot_(defect) is the total energy of the supercell containing the intrinsic defect in pristine or C-doped α-Al_2_O_3_, *E*_tot_(perfect) is the total energy of perfect Al_2_O_3_ supercell, μ_*i*_ is the chemical potentials of species *i* (Al, O, C), and *n*_*i*_ is the number of atoms of species *i* added to or removed from the perfect supercell to create the defect.

The calculated Frenkel formation energies for α-Al_2_O_3_, summarized in Table 3, are analyzed alongside the available experimental and theoretical results. The formation energy for an Al Frenkel pair is determined to be 5.75 eV, which is slightly higher (by 0.8 eV) than the experimental enthalpy per defect reported by Mohapatra and Kroger.^17^ Furthermore, the calculated Al Frenkel pair energy is lower than that of the O Frenkel defect. Catlow et al.^18^ reported using pair potentials that Al Frenkel pairs (FP_Al_) have higher formation energies than oxygen Frenkel pairs (FP_O_) in pure α-Al_2_O_3_, conflicting with our DFT result (FP_Al_: 5.75 eV vs FP_O_: 9.97 eV). Similar findings were also reported by molecular dynamics (MD) simulations.^19–21^ However, our findings align with Katsuyuki et al.^22^ and Koller et al.,^23^ who also observed lower Al Frenkel formation energies using DFT. The discrepancy with Catlow’s pair potential approach likely stems from its inability to capture electronic effects, whereas DFT accounts for covalent bonding and defect interactions.

**Table 3:** Calculated Frenkel Formation Energies (eV) in α-Al_2_O_3_ Presented together with the Available Experimental and Theoretical Data

*E*_form_	FP_O_	FP_Al_
Our Cal.	9.97	5.75
DFT Cal.^23^	9.30	4.10
DFT Cal.^22^	6.52	4.95
MD Cal.^18^	8.27	7.09
MD Cal.^18^	4.06	6.47
MD Cal.^19^	7.00	10.0
MD Cal.^20^	5.79	6.30
MD Cal.^21^	4.87	6.59
Exp.^17^		4.45

Defect formation energies for α-Al_2_O_3_, both with and without C doping, under O-poor and O-rich conditions, are summarized in Table 4. With the exception of the O interstitial (O_i_) in the α-Al_2_O_3_+C_Al_ system, the formation energies for intrinsic defects in C-doped α-Al_2_O_3_ are consistently lower than those in undoped α-Al_2_O_3_. This indicates that C doping generally reduces the formation energies of intrinsic defects, enhancing their likelihood of formation compared to pure material. Unlike the earlier study, which suggested carbon plays a minimal role in TL properties,^8^ our calculations show C doping reduces formation energies of intrinsic defects (V_Al_, O_i_, FP_Al_) by 10–30% compared to pure α-Al_2_O_3_ (Table 4). Yang et al. used thermoluminescence and optically stimulated luminescence (OSL) to show that carbon doping enhances α-Al_2_O_3_’s sensitivity by increasing defect concentrations.^5^ Our calculation (Table 4) shows C doping reduces defect formation energies (*e*.*g*., V_Al_: −8.87 eV vs −3.54 eV in pure α-Al_2_O_3_ under O-poor conditions), which aligns with Yang’s observation of more defects in doped samples.^5^ Under both O-poor and O-rich conditions, the formation energies for Al vacancy, O vacancy, Al interstitial, and O interstitial in α-Al_2_O_3_+C_O_ are lower than those in α-Al_2_O_3_+C_Al_. Conversely, the formation energies for Al Frenkel and O Frenkel are lower in α-Al_2_O_3_+C_Al_ compared to those in α-Al_2_O_3_+C_O_. Figure 2 illustrates the variation in formation energies for intrinsic defects in both pure and C-doped α-Al_2_O_3_ under the O-poor and O-rich conditions. As shown in Figure 2a, *E*_form_ for O_i_ and V_Al_ decreases as the oxygen chemical potential (μ_O_) shifts from O-rich to O-poor, while *E*_form_ for Al_i_ and V_O_ increases. For pure α-Al_2_O_3_, the defect formation energies follow the order V_Al_ < O_i_ < FP_Al_ < FP_O_ < Al_i_, indicating the stability sequence V_Al_ < O_i_ < FP_Al_ < FP_O_ < Al_i_. The formation energy of V_O_ is lower than that of FP_O_ under the O-rich conditions but higher under the O-poor conditions. Furthermore, V_O_ and V_Al_ are more readily formed under O-rich and O-poor conditions, respectively, consistent with the findings of Ao et al.^10^ Figure 2b,c shows similar μ_O_-dependent trends and relative stability orders for intrinsic defects in α-Al_2_O_3_+C_Al_ and α-Al_2_O_3_+C_O_ as observed in pure α-Al_2_O_3_. Notably, among all defects considered in both pure and C-doped systems, the Al vacancy (V_Al_) exhibits the lowest formation energy and is the most stable defect under the O-rich conditions.

**Figure 2:**
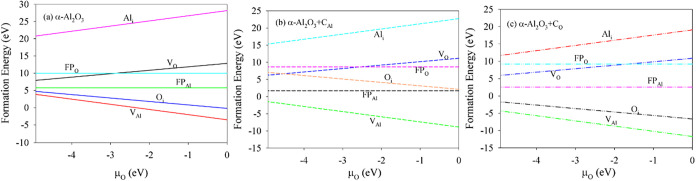
Variation of defect formation energies for (a) α-Al_2_O_3_, (b) α-Al_2_O_3_+C_Al_, and (c) α-Al_2_O_3_+C_O_ with intrinsic defects under O-poor and O-rich conditions.

**Table 4:** Defect Formation Energies (*E*_form_) for α-Al_2_O_3_ with and without C Doping under O-Poor and O-Rich Conditions

*E*_form_ (eV)	V_Al_	V_O_	Al_i_	O_i_	FP_Al_	FP_O_
O-poor condition						
α-Al_2_O_3_	–3.54	12.85	28.18	–0.21	7.35	9.97
α-Al_2_O_3_: C_Al_	–8.87	11.18	22.72	2.13	1.70	8.68
α-Al_2_O_3_: C_O_	–11.71	10.85	19.06	–6.65	2.54	9.21
O-rich condition						
α-Al_2_O_3_	3.86	7.92	20.79	4.72	7.35	9.97
α-Al_2_O_3_: C_Al_	–1.48	6.25	15.32	7.06	1.70	8.68
α-Al_2_O_3_: C_O_	–4.31	5.92	11.67	–1.72	2.54	9.21

### 3.3 Electronic Properties of Intrinsic Defects in Undoped and C-Doped α-Al_2_O_3_

The preceding calculations indicate that α-Al_2_O_3_ and C-doped α-Al_2_O_3_ systems containing aluminum vacancies (V_Al_), oxygen interstitials (O_i_), and Al Frenkel defects (FP_Al_) exhibit lower formation energies compared with other configurations. To elucidate the origin of these energetic differences, we analyze the total and partial density of states (DOS) distributions, presented in Figures 3–6. The Fermi level is set to 0 eV for all calculations. For comparison, the DOS of pristine α-Al_2_O_3_ and systems with C occupying Al-sites (α-Al_2_O_3_:C_Al_) or O sites (α-Al_2_O_3_:C_O_) are examined first (Figure 3). Pristine α-Al_2_O_3_ exhibits a calculated band gap (*E*_g_) of 6.16 eV, consistent with previous theoretical studies^10,15,22^ but smaller than the experimental value of 8.7 eV.^24^ This underestimation is typical of standard DFT calculations.^25,26^ The valence band maximum (VBM) is dominated by O 2*p* states, with minor contributions from Al 3s and 3p states. The conduction band minimum (CBM) comprises a hybridized state of O 2s, a state of O 2p, a state of Al 3s, and a state of Al 3p. These features agree well with prior theoretical reports.^27–29^ Substituting C for an Al site (α-Al_2_O_3_:C_Al_) significantly alters the DOS (Figure 3b): the valence band splits, and a distinct peak emerges near the VBM. An inset in Figure 3b magnifies this peak, revealing its composition primarily of the O 2p and C 2p states, indicative of the O–C covalent bonding. The CBM shifts to lower energies, resulting in a drastically reduced E_g_ of approximately 2.47 eV. The band gap narrowing we observe (*e*.*g*., 6.16 eV → 2.47 eV for C–Al doping) shifts the optical absorption edge to the visible range, which is experimentally observed in α-Al_2_O_3_:C.^5^ This visible absorption is critical for OSL, where optical stimulation releases trapped charges, enabling repeated readout—a key advantage over thermoluminescence. For C substitution for an O site (α-Al_2_O_3_:C_O_, Figure 3c), several small peaks attributed to hybridized O 2p/2s and C 2p/2s states appear near the VBM. The CBM also shifts downward, yielding a calculated *E*_g_ of 4.45 eV. Zhu et al. predicted that C substitution at Al sites narrows the band gap, but did not quantify the effect.^9^ Our work extends this by showing that C–Al substitution reduces the gap by ∼3.7 eV (from 6.16 to 2.47 eV), while C–O substitution narrows it by only ∼1.7 eV (to 4.45 eV). This site-dependent gap reduction is critical for tailoring the optical properties.

**Figure 3:**
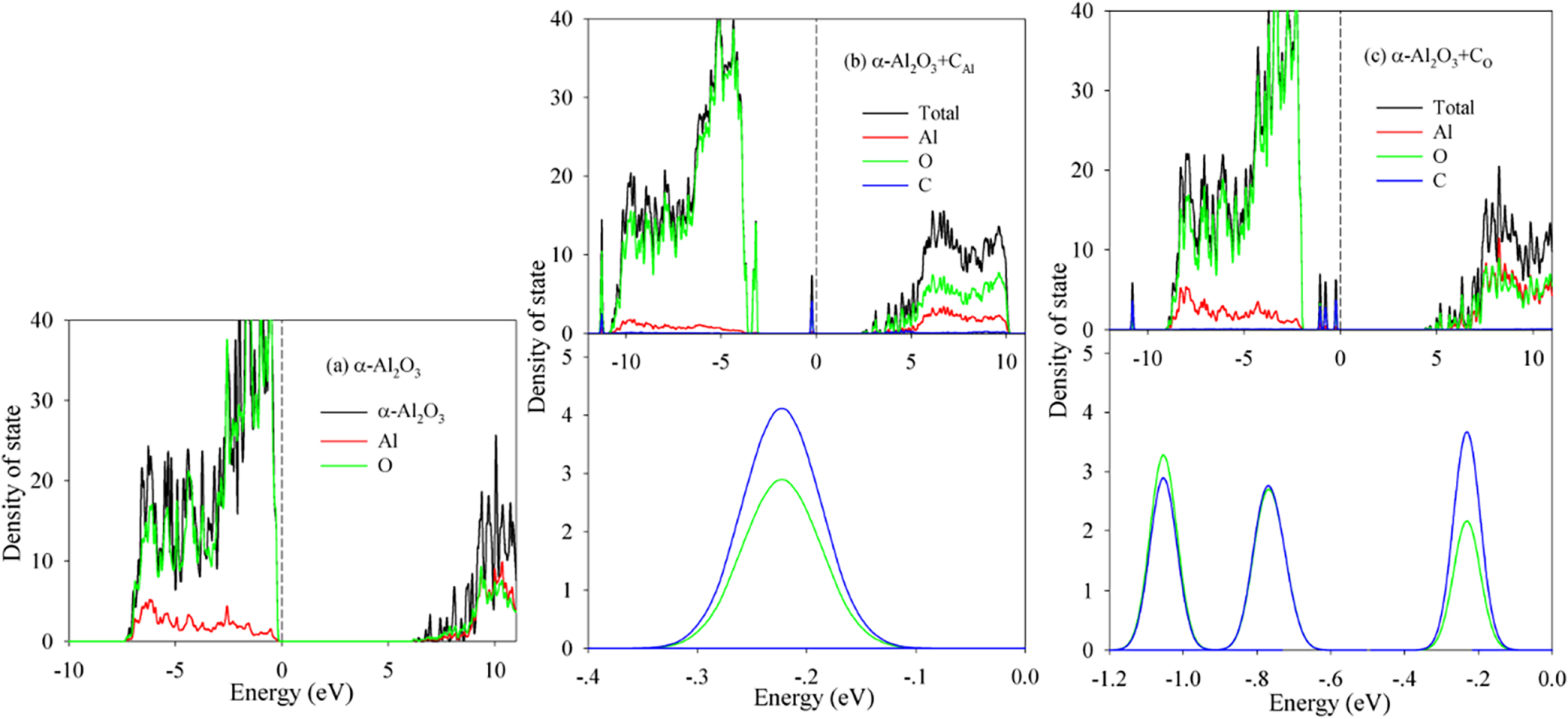
Density of state distribution for (a) α-Al_2_O_3_; (b) α-Al_2_O_3_+C_Al_; and (c) α-Al_2_O_3_+C_O_.

Figure 4a displays the total DOS values for the α-Al_2_O_3_:V_Al_ system. The distribution closely resembles that of pristine α-Al_2_O_3_, with a calculated band gap of 6.22 eV. When a C atom occupies an Al site within the α-Al_2_O_3_:V_Al_ system (Figure 4b), two defect states emerge: (i) a state at the Fermi level, primarily consisting of an O 2*p* character with minor O 2s contribution; (ii) a state between approximately 4.0 and 4.3 eV, originating from hybridized O 2s/2p and Al 3s/3p states. For C substitution for an O site in α-Al_2_O_3_:V_Al_ (Figure 4c), multiple defect states appear near the valence and conduction band edges, reducing *E*_g_ to 2.68 eV. Specifically, states in the ranges of −3.0 to −2.4 eV and 2.6 to 4.5 eV are dominated by the O 2p character, with a minor contribution from C 2p/2s hybrid states. States spanning −0.4 to 0 eV primarily arise from C 2p/2s orbitals hybridized with the O 2p/2s states.

**Figure 4:**
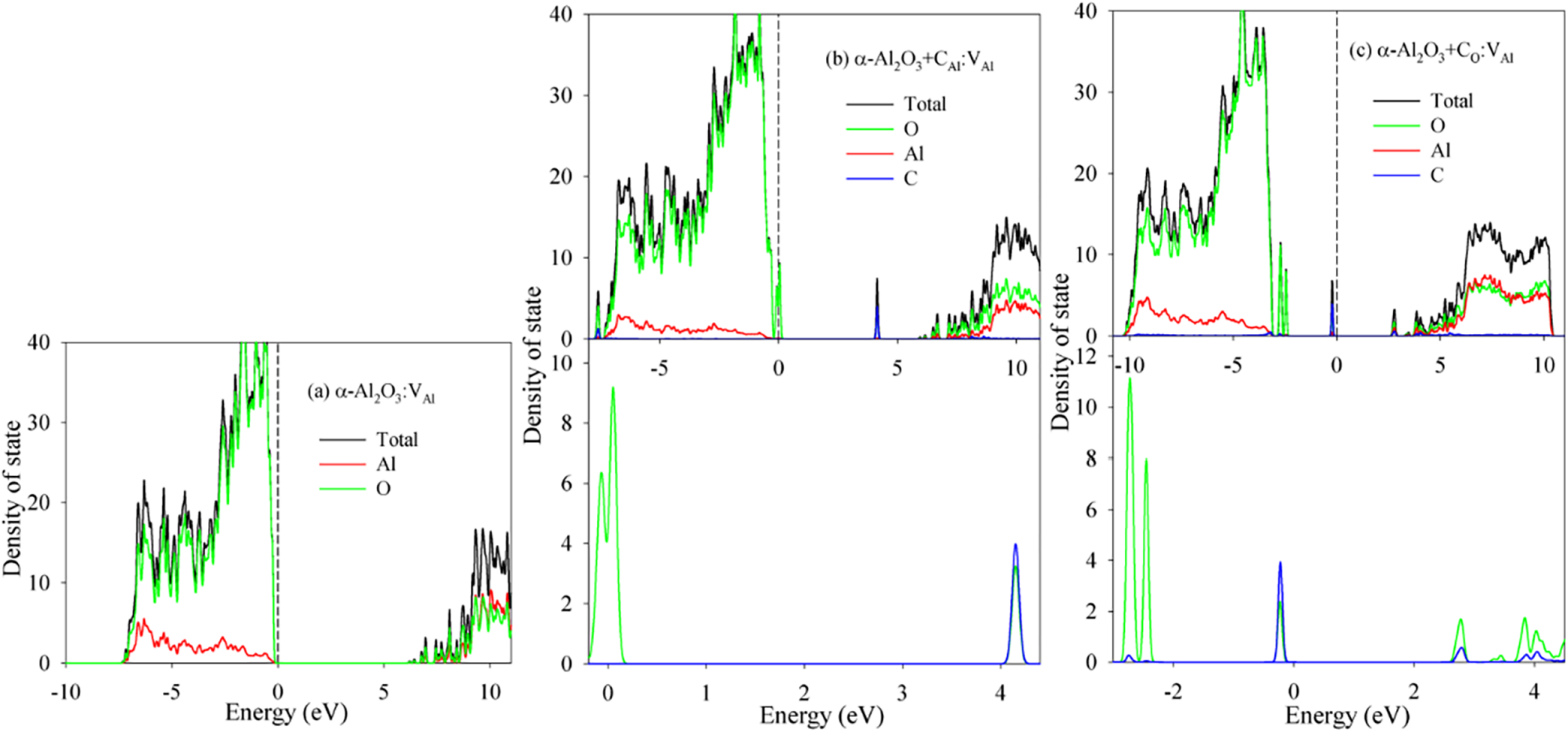
Density of state distribution for (a) α-Al_2_O_3_:V_Al_; (b) α-Al_2_O_3_+C_Al_:V_Al_; and (c) α-Al_2_O_3_+C_O_:V_Al_.

In the α-Al_2_O_3_:O_i_ system, the O interstitial induces defect states near the VBM, primarily composed of O 2p orbitals with minor contributions from Al 3*s*/3*p* states, as shown in Figure 5a. This defect also causes a slight downward shift of CBM, reducing the band gap by 1.1 eV compared with pristine α-Al_2_O_3_. Substituting a C atom for an Al atom within this α-Al_2_O_3_:O_i_ system introduces an impurity state at the CBM (Figure 5b), resulting in an *E*_g_ of 1.60 eV. Conversely, substituting C for an O site in the α-Al_2_O_3_:O_i_ system generates impurity states near the VBM, dominated by hybridized C 2p and O 2p character (Figure 5c), yielding a calculated *E*_g_ of 4.41 eV.

**Figure 5:**
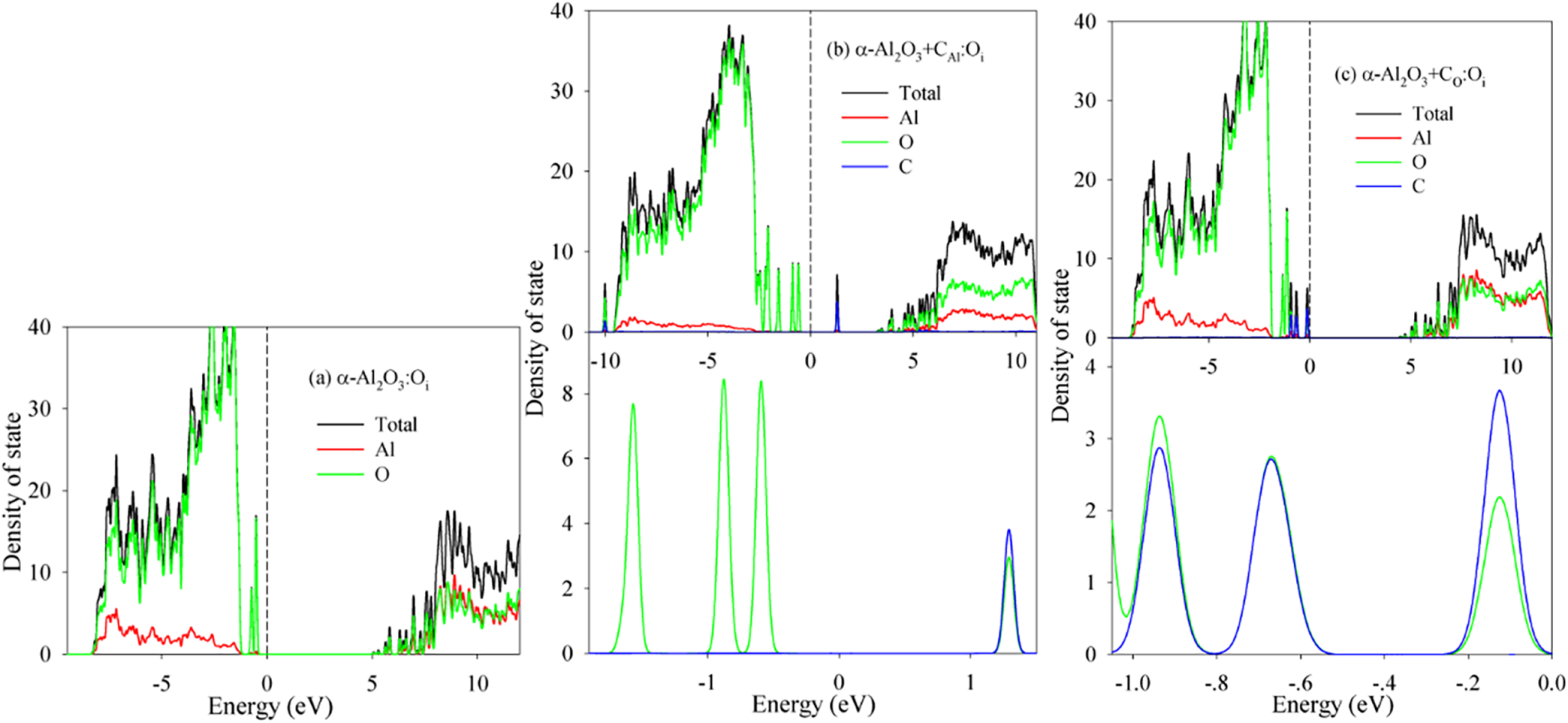
Density of state distribution for (a) α-Al_2_O_3_:O_i_; (b) α-Al_2_O_3_+C_Al_:O_i_; and (c) α-Al_2_O_3_+C_O_:O_i_.

In the α-Al_2_O_3_:FP_Al_ system, some O 2p states appear at the VBM and cause a slight downward shift of the CBM relative to pristine α-Al_2_O_3_ (Figure 6a). This reduces the band gap by 1.12 eV. When C is substituted for an Al site in this FP_Al_-containing system (α-Al_2_O_3_+C_Al_:FP_Al_), an impurity state emerges at the VBM (Figure 6b), primarily composed of hybridized C 2p and O 2p states. An additional downward shift of the CBM results in a reduced *E*_g_ of 2.11 eV. For C substitution for an O site within the FP_Al_ system (α-Al_2_O_3_+C_O_: FP_Al_), three distinct impurity states dominated by C 2p and O 2p character appear near the VBM (Figure 6c). The conduction band density of states resembles that of the α-Al_2_O_3_+C_Al_:FP_Al_ system, obtaining an *E*_g_ of 2.60 eV.

**Figure 6:**
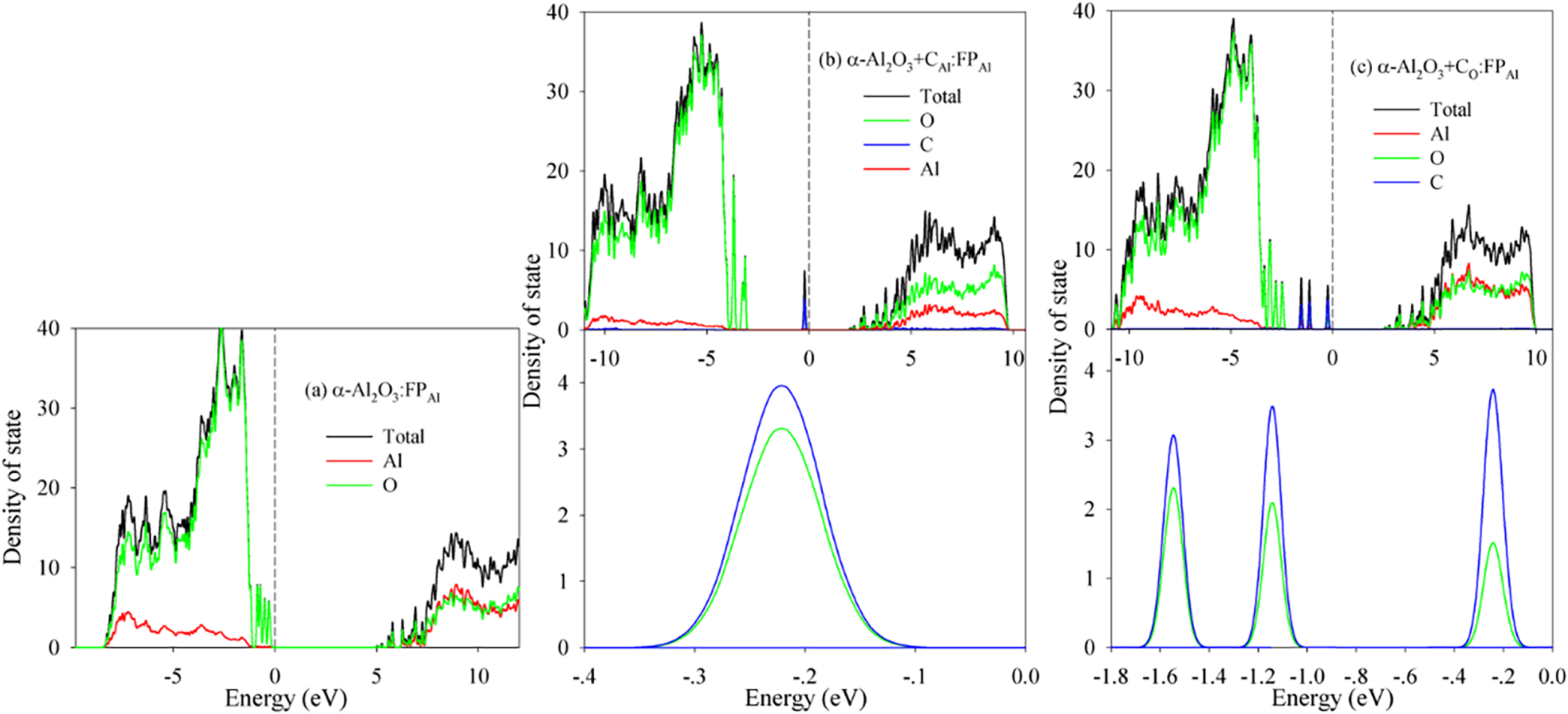
Density of state distribution for (a) α-Al_2_O_3_:FP_Al_; (b) α-Al_2_O_3_+C_Al_:FP_Al_; and (c) α-Al_2_O_3_+C_O_:FP_Al_.

Collectively, the DOS analysis elucidates the electronic origins underlying the reduced formation energies of aluminum vacancies (V_Al_), oxygen interstitials (O_i_), and Al Frenkel pairs (FP_Al_) in C-doped α-Al_2_O_3_. C doping systematically narrows the band gap across all defective systems, primarily through the introduction of impurity states near the band edges. These states predominantly arise from hybridization between C 2p/2s and host O 2p/2s orbitals, with the extent of band gap reduction exhibiting strong dependence on the C incorporation site (Al vs O substitution). Notably, C substitution at Al sites induces more pronounced gap narrowing compared with that of O-site substitution. The formation of these midgap states, particularly those involving C–O hybridization, likely facilitates charge compensation and stabilizes intrinsic defects, thereby rationalizing their lower formation energies in C-doped alumina.

## 4 Conclusions

The influence of C doping on α-Al_2_O_3_ containing intrinsic defects was systematically investigated by using density functional theory (DFT) calculations. Structural, energetic, and electronic properties were comprehensively analyzed for both C-doped and undoped α-Al_2_O_3_ systems with various intrinsic defects (V_Al_, V_O_, A_i_, and O_i_ FP_Al_ and FP_O_). Key findings reveal that C substitution for Al sites induces minimal structural deformation in defect-containing α-Al_2_O_3_, while C doping at the O sites results in significantly greater lattice distortion. Analysis of average bond distances between defect sites and neighboring atoms demonstrates distinct spatial effects. For V_Al_- and A_i_-containing systems, C incorporation predominantly alters O-atom coordination distances, whereas for V_O_- and O_i_-containing configurations, Al-atom coordination distances exhibit more pronounced changes compared to O-atom interactions. Notably, the formation energies of intrinsic defects in C-doped α-Al_2_O_3_ systems are consistently lower than those in the pure state, indicating an enhanced defect formation propensity under C doping. Electronic structure calculations further show that C incorporation systematically reduces the band gaps in pristine α-Al_2_O_3_ and in systems containing aluminum vacancies, O interstitials, and Al Frenkel pairs. These findings demonstrate that carbon doping offers a promising pathway to simultaneously tune the defect stability, structural characteristics, and electronic band structure of α-Al_2_O_3_, potentially enhancing its functional performance in application**s** such as catalysis or optoelectronics.
